# Persistent Atrium Standstill Post Atrial Fibrillation Ablation Therapy

**DOI:** 10.7759/cureus.25293

**Published:** 2022-05-24

**Authors:** Maya A Khatoun, Houssein Toufayli, Mary-Joe Touma, Samer R Nasr

**Affiliations:** 1 Cardiology, University of Balamand, Beirut, LBN; 2 Cardiology, Mount Lebanon Hospital, Beirut, LBN

**Keywords:** enlarged left atrium, direct cardioversion, ablation therapy, atrial standstill, atrial fibrillation

## Abstract

Atrial standstill is a rare condition in which the atrium loses its mechanical contraction with or without losing the electrical conduction. In this report, we discuss a case of a 64-year-old male patient with a history of hypertrophic cardiomyopathy (HCM) and persistent refractory atrial fibrillation (AF). He underwent ablation therapy with a successful return to sinus rhythm. However, post-procedure echocardiography imaging showed the absence of left atrium mechanical activity. We aim to highlight the importance of assessing atrial mechanical activity by imaging after sinus cardioversion in order to treat any preventable complications promptly.

## Introduction

Atrial fibrillation (AF) is the most common type of arrhythmia, and it can lead to devastating complications if untreated [1,2]. Cardiovascular disease is an important risk factor for AF development [2]. AF is frequent in adult patients with hypertrophic cardiomyopathy (HCM; 2% incidence of new cases annually), resulting in a greater risk for AF development compared to the general population [3,4]. Longstanding AF may cause an atrial standstill [5]. Atrial standstill is a condition, involving one or both atria, characterized by an impairment of electrical function or mechanical function (detected by imaging). It may be either transient or permanent, partial or total [5,6]. The pathophysiology of atrial standstill is mostly related to the loss of the atrial myocardium with replacement by fibrofatty tissue, consistent with atrial myopathy [7]. Atrial standstill can be idiopathic, primary, or secondary. In the primary persistent form, atrial inactivity is accompanied by atrial dilation and thrombotic complications [7]. We discuss a case of a 64-year-old male patient with a history of hypertrophic obstructive cardiomyopathy (HOCM) and persistent AF, status post-primary prevention pacemaker/implantable cardioverter-defibrillator (ICD) for severe septal hypertrophy (35 mm at the time of implant). The patient underwent several electrical cardioversions and two ablation trials and was found to have a persistent isolated left atrial standstill.

## Case presentation

**Figure 1:**
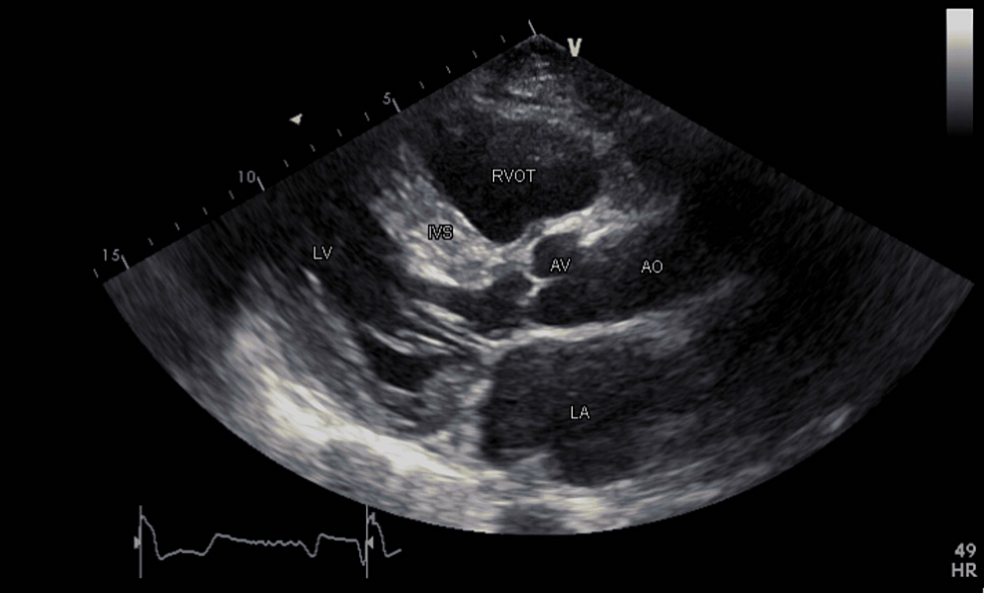
Parasternal long-axis view showing interventricular septal hypertrophy and dilated left atrium RVOT: right ventricle outflow tract; AO: aorta; AV: aortic valve; LV: left ventricle; LA: left atrium; IVS: interventricular septum

**Figure 2:**
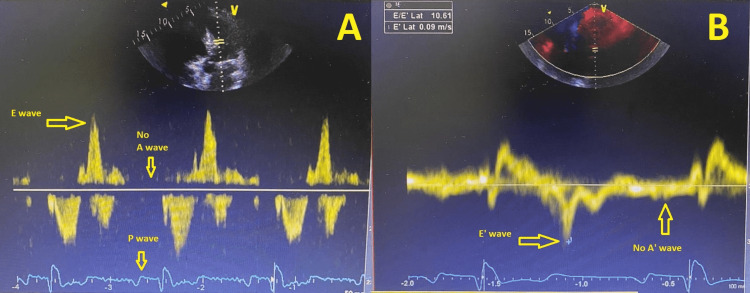
Pulsed-wave Doppler recording of the mitral inflow and tissue Doppler at the mitral annulus A: pulsed-wave Doppler recording of the mitral inflow showing the presence of P wave on ECG, E wave, and the absence of A wave; B: tissue Doppler at the mitral annulus showing the presence of E' wave without A' wave P wave: electrical conduction of the left atrium; ECG: electrocardiogram; E wave: peak velocity blood flow from the left ventricle relaxation in early diastole; A wave: peak velocity flow in late diastole caused by atrial contraction; E' wave: early diastolic filling of the left ventricle; A' wave: late diastolic filling caused by atrial contraction

The patient was a 64-year-old male diagnosed with AF at the age of 45. The echocardiography at that time had shown severe HOCM with no gradient (Figure 1). He had undergone pacemaker/ICD placement for severe septal hypertrophy (35 mm at the time of implant) as a primary preventive measure against sudden cardiac death. He had remained refractory to medical therapy despite several electrical cardioversion attempts. He had complained of dyspnea and palpitations and had consequently undergone his first ablation at the age of 59, which had been repeated at the age of 62 due to recurrence of ablation. Post-procedure transthoracic echocardiogram (TTE) assessment had shown the restoration of left atrium electrical conduction without the return of atrial mechanical activity as documented by pulsed-wave Doppler imaging of the mitral inflow and tissue Doppler imaging (TDI) at the mitral annulus (Figure 2).

**Figure 3:**
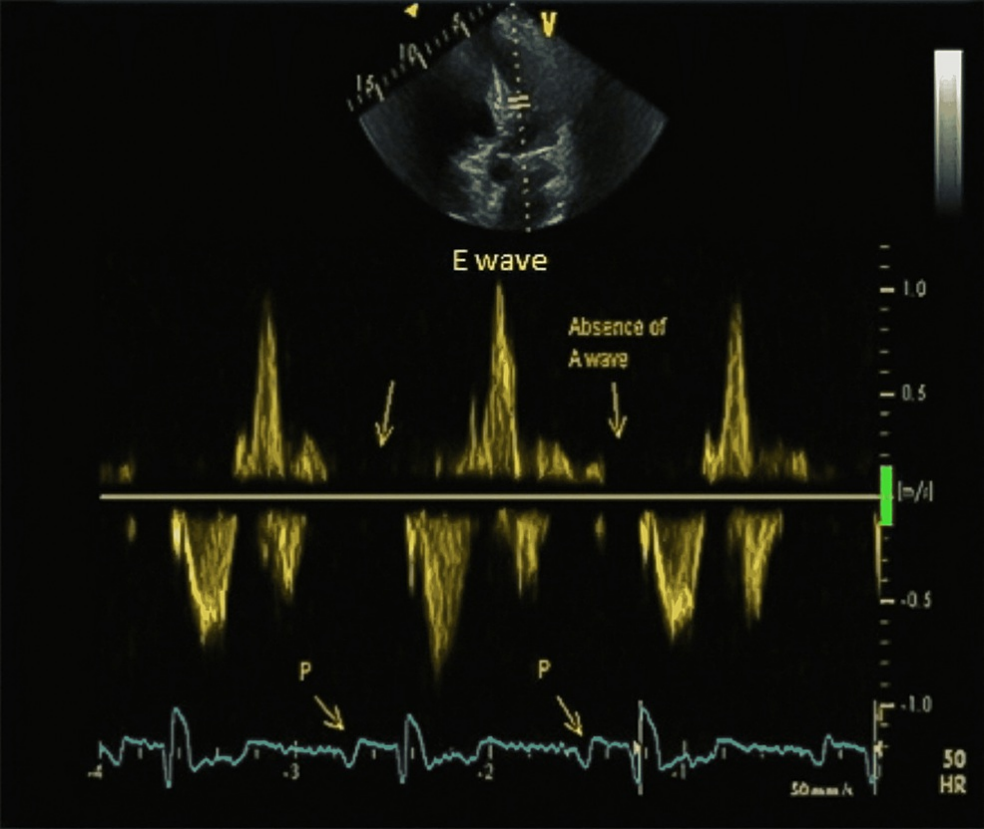
Pulsed-wave Doppler TTE showing the presence of E wave velocity with the absence of A wave (atrial contraction indicated by arrow) TTE: transthoracic echocardiogram

**Figure 4:**
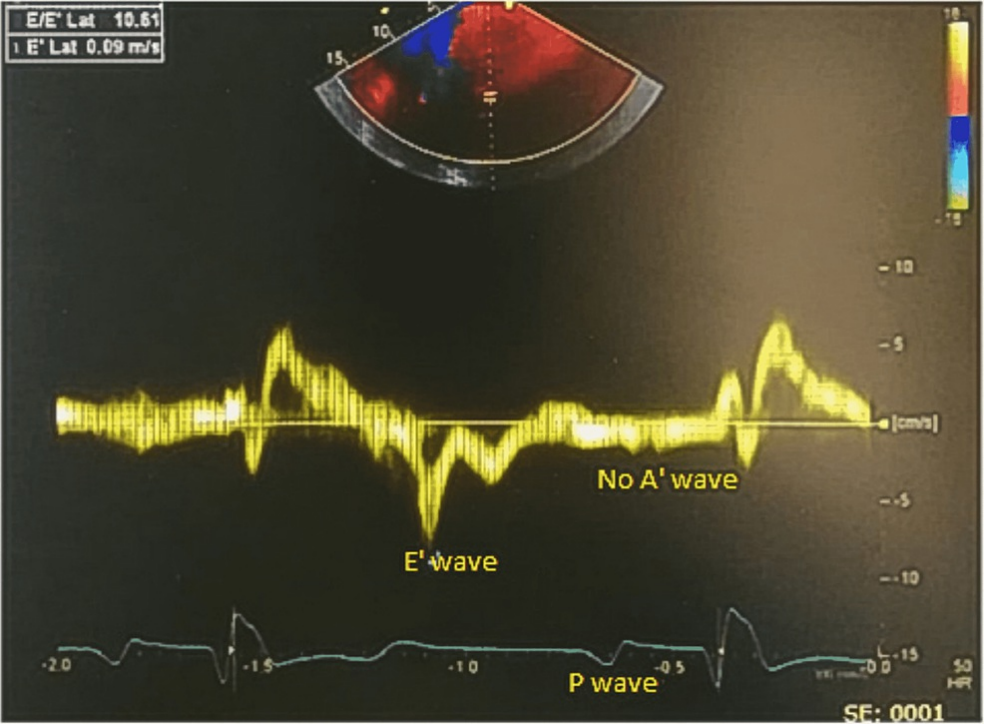
Tissue Doppler showing the presence of E’ wave and P wave but the absence of A’ wave

The patient presented for thromboembolic risk re-evaluation and possible discontinuation of anticoagulation therapy after successful ablation therapy. He was found to be in sinus rhythm and asymptomatic. Vital signs were within the normal range: temperature of 37.5 °C, heart rate of 85 beats per minute, and blood pressure of 126/75 mmHg. He had no complaints of dyspnea or palpitations and had a good functional capacity. Upon physical examination, his heart sounds were regular. A 2D TTE showed a dilated left atrium and the presence of preserved atrial electrical activity (P wave on electrocardiogram) (Figures 1-3). Pulsed-wave Doppler TTE showed the presence of E wave velocity with the absence of A wave (atrial contraction indicated by the arrow in Figure 3). Tissue Doppler showed the presence of E’ wave, but the absence of A’ wave (Figure 4); It should be noted that an echocardiogram is one of the most useful methods to assess cardiac chambers’ structure and function, including left atrial contractility reflected by A wave (peak velocity flow in late diastole caused by atrial contraction on pulsed-wave Doppler), and A’ wave, which reflects late diastolic filling of the left ventricle caused by atrial contraction on tissue Doppler TTE at the mitral annulus.

Accordingly, our decision was to keep him on anticoagulation therapy until further information about the management of persistent atrial standstill disease was available.

## Discussion

Atrial standstill is a rare condition where either the right or the left atrium loses its electrical excitability. It is characterized by an electrical or mechanical standstill [8]. In its transient form, It has been reported following several conditions including hyperkalemia, drug toxicity (digoxin, quinidine, and verapamil), cardiac ischemia, cardioversion, and cardiac surgeries [9,10]. None of these conditions was evident in our case. A persistent form of atrial standstill manifests in the form of syncope or peripheral embolism [11].

Several cases of isolated left atrium standstill have been reported in patients with AF and HCM. Some of them underwent left atrium ablation while others underwent current cardioversion. However, all the cases were diagnosed with atrium standstill while in sinus rhythm [12]. The main imaging techniques used to detect this condition are transthoracic echocardiography by assessing the pulsed-wave Doppler imaging of mitral valve (MV), TDI of the lateral and septal MV annulus, and pulsed-wave Doppler of pulmonary vein (PV) flow [12]. Our case showed the absence of left atrium mechanical activity confirmed by the absence of A wave velocity normally caused by atrial contraction on pulsed-wave Doppler imaging of MV, despite the maintenance of electrical atrial activity on the electrocardiogram as mentioned before. Also, TDI showed the absence of A’ wave and confirmed the persistent loss of mechanical left atrial contraction. This condition was detected post-ablation procedure and persisted during patient follow-up after two years.

The incidence rate of left atrium standstill is still unknown and its pathologic basis is not well understood [12]. However, it seems that long persistent AF, especially in hypertrophic cardiomyopathy, results in atrial fibrosis, and left atrium ablation therapy also enhances atrial fibrosis and impairs its contractility [12].

The main concern regarding atrial standstill is that it exposes patients to an increased risk of embolic events caused by blood stasis due to atrial dysfunction and dilation [10]. In case of atrial standstill, patients should be on lifelong oral anticoagulation in order to prevent thromboembolic events, especially cerebral ischemia, thereby avoiding deleterious sequelae [13,14].

## Conclusions

In ablation therapy for AF, it is critical to assess left atrial contractile function before and after the procedure through detailed echocardiographic studies of the left atrium. The pathophysiology and clinical outcomes of the left atrium standstill remain unclear as of now. Hence, further research is needed to better understand the impact of the absence of mechanical contraction, in the presence of electrical conduction, on thromboembolic events, mortality rates, and heart failure risks in order to treat preventable complications.
